# Alterations in transcranial sonography among Huntington’s disease patients with psychiatric symptoms

**DOI:** 10.1007/s00702-020-02187-x

**Published:** 2020-04-13

**Authors:** Grzegorz Witkowski, Katarzyna Jachinska, Iwona Stepniak, Karolina Ziora-Jakutowicz, Halina Sienkiewicz-Jarosz

**Affiliations:** 1 I-st Department of Neurology, Institute of Psychiatry and Neurology, Sobieskiego 9 Str., 02-957 Warsaw, Poland; 2 Department of Genetics, Institute of Psychiatry and Neurology, Warsaw, Poland

**Keywords:** Huntington’s disease, Transcranial sonography, Nucleus raphe, Substantia nigra, Depression

## Abstract

Transcranial sonography (TCS) is a diagnostic tool in mood and movement disorders. Alterations within the raphe mesencephalic nucleus in the brain have been reported not only in patients with major depression but in patients with depressive symptoms accompanying several neurodegenerative disorders. The aim of the study was to assess the echogenicity of the nucleus raphe and other basal ganglia in patients with Huntington’s disease (HD). TCS was performed in 127 HD patients participating in observational studies (Registry/Enroll-HD) in the Institute of Psychiatry and Neurology (Warsaw, Poland). Raphe hypoechogenicity was found in 78% of HD patients with current symptoms of depression (according to DSM-IV criteria), 57% of patients with a previous history of depression, and 56.8% patients who lacked signs or history of depression. Patients with hypoechogenic raphe reported significantly higher depression as measured on the BDI (15.6 ± 1.7) as compared to patients with normal echogenicity (9.5 ± 1.2), (*p* = 0.023). The diameter of the third ventricle was negatively correlated with Mini-Mental State Examination (MMSE) (rho − 0.37) and total functional capacity (TFC) scores (rho − 0.26). Hyperechogenic substantia nigra was visualized in 66,4% patients with HD and the degree of hyperechogenicity was correlated with the total motor score (TMS) (rho − 0.38). Changes in echogenicity of the basal ganglia are related to both depressive and motor symptoms among patients with HD.

## Introduction

In recent years, transcranial sonography (TCS) has been used to visualize structural abnormalities in the brain parenchyma in patients with movement disorders. TCS is a sensitive diagnostic tool in movement disorders, particularly for differential diagnosis of parkinsonian syndromes (Walter et al. 2007). Hyperechogenicity of substantia nigra is a characteristic of Parkinson’s disease (PD), wherein atypical parkinsonian syndromes like multiple system atrophy with parkinsonian phenotype (MSA-P) and progressive nuclear palsy (PSP), hyperechogenicity of the lentiform nuclei (LN) is observed more frequently (for recent metaanalysis see Richter et al. 2020). Enlarged diameter of the third ventricle is a finding that is more typical for progressive supranuclear palsy (PSP). Both substantia nigra and lentiform nuclei hyperechogenicities are found in Wilson’s disease (Svetel et al. 2012).

Studies of TCS report changes in the echotexture of serotoninergic mesencephalic raphe nucleus in patients with major depression and depressive symptoms in several neurodegenerative disorders. It has been established that reduced echogenicity of nucleus raphe correlates with signs and/or a history of depression in patients with affective syndromes, regardless of their type (i.e., positive TCS findings in up to 70% of patients with signs of depression and in up to 8% in healthy controls) (Mijajlovic 2012; Walter et al. 2007). These echogenicity changes were found to be independent of current antidepressant pharmacotherapy, time from symptom onset, age, and gender. There was also a correlation between the TCS findings and positive response to selective serotonin reuptake inhibitor (SSRI) pharmacotherapy (Mijajlovic 2012). A hypoechogenic nucleus raphe is also a frequent TCS finding among patients with major depression and depressive symptoms related to Parkinson’s disease (Richter et al. 2018).

Depression is the most frequent psychiatric symptom among patients with Huntington’s disease (HD) (Du et al. 2013). Depression is reported in 30–70% of presymptomatic HD carriers (Duff et al. 2007). The suicidal rate among patients with HD is 3–7.3% (Di Maio et al.^1993^).

To-date, three published studies have evaluated changes in basal ganglia echogenicity in HD patients (Saft et al. 2015; Krogias et al. 2010; Postert et al. 1999). These studies, however, were performed on limited groups of patients and revealed changes in echogenicity of both the substantia nigra and the nucleus raphe.

The primary goal of this study was to assess the frequency of changes in echogenicity of the basal ganglia and the brainstem raphe, and to correlate these changes with neurological and psychiatric status in a large cohort of HD patients in different stages of the disease.

## Methods

### Participants

We recruited patients with genetically confirmed HD and who: (1) were participating in the Registry observational study; and (2) undergone annual visits at the study site in the Institute of Psychiatry and Neurology (Warsaw, Poland). The Registry was the European observational study governed by the European Huntington Disease Network (EHDN). Basic demographic data (i.e., age, age at onset of motor symptoms, and depression), drugs used, and comorbidity were recorded. For the study data, we used the results of standard assessments performed during annual Registry visits, including UHDRS total motor score (TMS), total functional capacity (TFC), together with Mini-Mental State Examination (MMSE) done as a standard assessment during a patient visit in an outpatient clinic. All patients were also asked to complete the Beck Depression Inventory (BDI), which was a part of the aforementioned Registry version 2.0 observational study protocol (Handley et al. 2011). Patient medical record data, available via the Department of Genetics of our institution, were reviewed to assess previous psychiatric history. Diagnosis of depression was based on DSM-IV criteria.

TMS was considered to be significant when the score was higher than 5, with a level of 4 for diagnostic confidence. The results of BDI were found to be consistent with moderate to severe depression when the score was higher than 18 (Beck et al. 1988).

The staging of the disease was based on TFC values: stage 1, TFC 13–11; stage 2, 10–7; stage 3, 6–3; stage 4, 1–2 pts (Paulsen et al. 2010).

Moreover, we recruited a group of 84 age-matched non-HD controls. Controls were individuals without a history of psychiatric or other neurological disorder.

All clinical assessments and the review of medical history were done by investigators certified in the use of the UHDRS scale with more than 5 years of experience in HD (I.S, K.Z-J). The TCS study protocol was approved by the local Institutional Review Board, as well as, the EHDN Scientific Bioethical and Advisory Committee (SBAC). Informed consent was obtained from all participants—both HD subjects and non-HD controls.

### Transcranial sonography

TCS was performed and assessed by an experienced ultrasonographer who was blind to the clinical history of patients (K.J). TCS was performed through the transtemporal bone window using the Aloka ProSound Alpha 10 System (Hitachi Aloka Medical, Ltd). A second ultrasonographer (G.W.) made an additional assessment of the echogenicity of subcortical structures off-line based on saved TCS dicom images. Patients with the inadequate temporal bone window had to be excluded from the study. TCS was performed using a 2.5-MHz transducer with a depth of 14–16 cm. Brightness and time gain compensation were dependent on the sonographer (Berg et al. 2001). The protocol used was based on published recommendations for TCS (Walter et al. 2007; Walter and Skoloudik 2014).

**Fig. 1:**
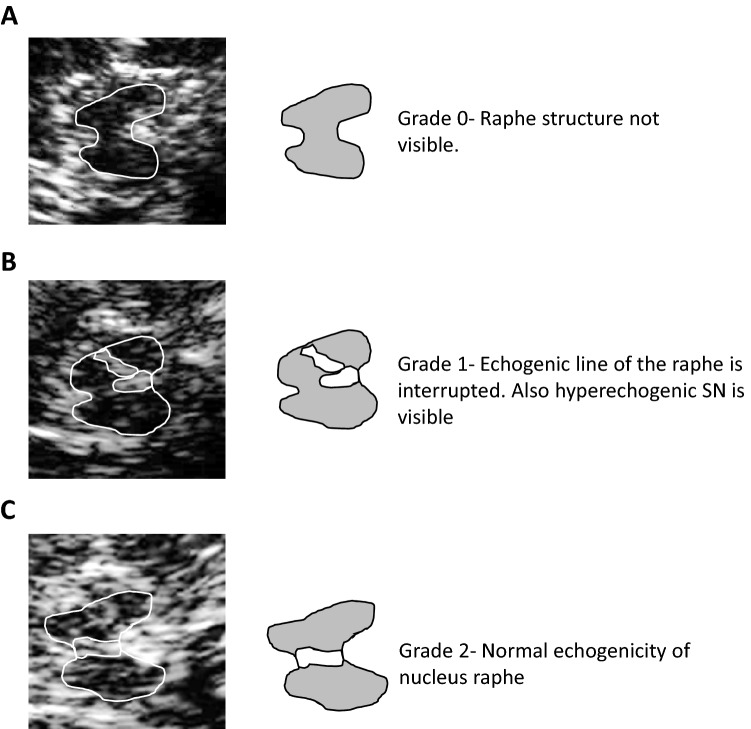
Examples of various TCS presentations of midbrain nucleus raphe

The midbrain and diencephalic examination planes were visualized. Echogenicity of the brain stem raphe was classified using a standard 3-point scale: 0 = raphe structure not visible, 1 = reduced echogenicity (i.e., the echogenic line of the brain stem raphe is interrupted or appears abnormally slight and thin), and 2 = normal echogenicity (3) (Fig. 1). The diameter of third ventricle was measured as the minimum distance between hyperechogenic ventricle walls (Wollenweber et al. 2011). Echogenicity of the substantia nigra was measured in axial sections. Sizes of less than 0.2 cm^2^ were defined as ‘normal’; sizes between 0.2 cm^2^ and 0.25 cm^2^ were classified as ‘moderate’, and sizes larger than 0.25 cm^2^ were defined as ‘markedly hyperechogenic’ (Walter et al. 2007; Walter and Skoloudik 2014; Krogias et al. 2011). The aforementioned structures were analyzed for both hemispheres (Krogias et al. 2011; Prestel et al. 2006).

### Statistical analysis

The basic analysis of demographic and clinical data was performed with the use of GraphPad Prism software (GraphPad, version 10). Means and proportions were compared using a parametric ANOVA or a non-parametric Kruskal–Wallis ANOVA, as appropriate. Spearman rho was applied for correlation analysis. Data were presented as mean ± standard error (SE), or as mean (range). The inter-rater agreement between radiologists assessing the echogenicity of nucleus raphe and substantia nigra was calculated using a reliability analysis (kappa statistics). If the rater’s assessments were not in agreement, a senior investigator (H. S-J.) made the final decision regarding the interpretation of a given image.

## Results

### Clinical demographics

Of the 139 patients tested, 11 were excluded due to an insufficient temporal bone window. The remaining group of 126 patients consisted of 66 women and 60 men. Demographic data for included participants and control group are presented in Table 1.

**Table 1:** Basic demographic and clinical data together with basic TCS findings of the HD patient and control group samples

	HD subjects	Control subjects
*n*	126	84
sex	66 female, 60 male subjects	30 female, 54 male subjects
Mean age	53 (range 29–71)	52.4 (range 27–70)
Larger allele	Range 39–63	
Smaller allele	Range 12–30	
Mean UHDRS TMS	37.6 (range 6–74)	
Mean TFC	8.5 (range 1–13)	
Mean MMSE	24.8 (range 11–30)	
Percentage of subjects with altered nucleus raphe echogenicity (score 0 or 1)	67.4% (85 out of 126 subjects)	21.4% (18 out of 84)
Mean diameter of the third ventricle	9.4 mm (range: 4–14.7 mm)	5.6 mm (range: 2.5–8.9 mm)
Percentage of subjects with altered echogenicity of the substantia nigra	66.4% (79 out of 119 subjects)	10.7% (9 out of 84 subjects)

### Huntington’s disease clinical characteristics

All HD patients presented with motor symptoms, with a mean UHDRS TMS score of 37.6 (range 6–74). The mean age of motor symptom onset was 42.9 ± 1.1 years. The mean MMSE was 24 (range 11–30). The stage of disease was assessed based on TFC scores. There were 31 patients in stage 1 (TFC score 11–13), 59 patients in stage 2 (TFC score 7–10), and 31 patients in stage 3 (TFC score 3–6).

61 HD patients had signs of depression and fulfilled the DSM-IV criteria for a major depressive episode. For 21 of those cases, depression was not currently present but was a significant part of the patient’s previous medical history. 65 HD patients described an earlier onset of depressive symptoms as compared to motor symptoms, with mean age of onset of depressive symptoms as 39.1 ± 1.4 years. 75 patients were taking antidepressants at the time of examination. In the group of patients with a current diagnosis of depression, the mean BDI score was 19.6 ± 0.7. In patients with a previous history of depression, the mean BDI score was 10.1 ± 0.8. For HD patients with neither a current nor prior history of depression, mean BDI score was 5.3 ± 0.7.

### TCS findings

**Fig. 2:**
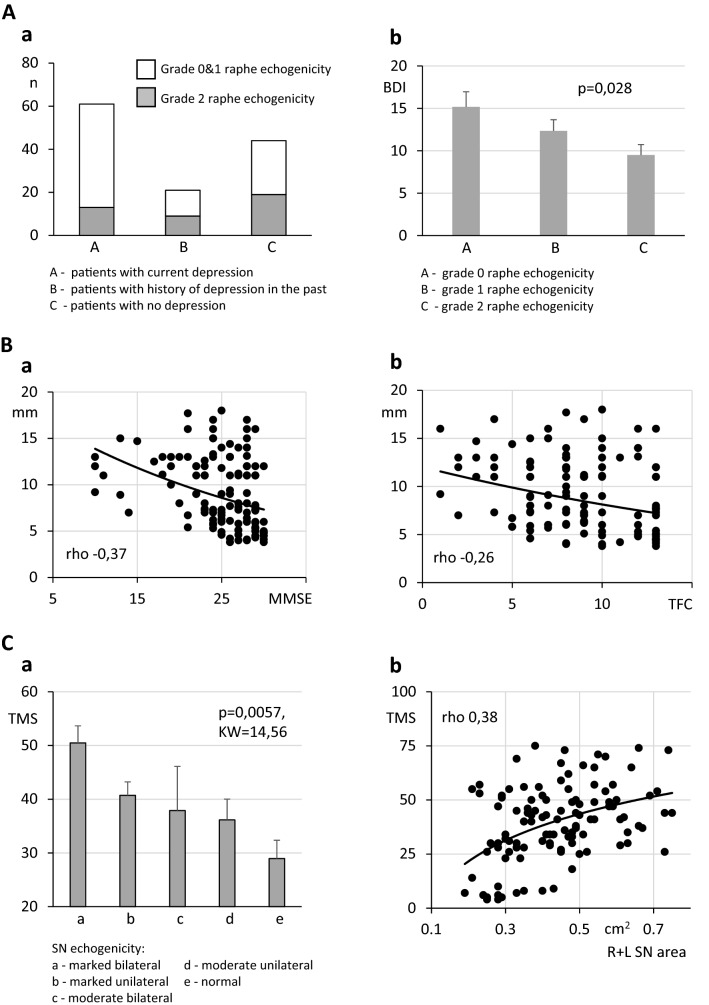
**Aa** Number of HD patients with and without abnormal nucleus raphe echogenicity in 3 subpopulations of patients: (1) patients with current signs of depression, (2) patients with depression in their past medical history, and (3) patients with neither present nor past depression. **Ab** BDI score in patients with different echogenicity of the nucleus raphe. **Ba** Spearman rho correlation between the diameter of the third ventricle (vertical axis) and MMSE score (horizontal axis). **Bb** Spearman rho correlation between the diameter of the third ventricle (vertical axis) and TFC score (horizontal axis). **Ca** UHDRS TMS scores in HD patients with different degrees of SN hyperechogenicity. **Cb** Spearman rho correlation between TMS scores (vertical axis) and the summarized area of the right and left SN (horizontal axis).
  We also found that patients with disturbed echogenicity had significantly higher BDI scores as compared to patients without disturbed echogenicity: grade 0-—BDI 15.1 ± 1.8, grade 1—BDI 12.3 ± 1.3, grade 2—BDI 9.5 ± 1.2 (Anova, *F* = 3.68, *p* = 0.028) (Fig. 2Ab). The Spearman correlation between BDI score and nucleus raphe echogenicity score was weak but significant (*r* = − 0.27, *p* = 0.002). In the group of patients taking antidepressants, the percentage of subjects with altered raphe echogenicity was 72% (i.e., 54 of 75 patients). In the group of patients currently not taking antidepressants, this percentage was lower but also significant (i.e., 57.7%, or 30 out of 52 patients).
  The raphe echogenicity assessment was characterized with a global sensitivity of 78% (95% CI 0.65–0.89), a specificity of 48% (95% CI 0.28–0.58), a positive predictive value (PPV) of 69% (95% CI 0.59–0.77), and a negative predictive value (NPV) of 30% (95% CI 0.22–0.40) in the prediction of HD patients with current signs of depression.
  In the control group, abnormal raphe echogenicity (i.e., grade 0 or 1) was found in 21.4% of participants (i.e., 18 out of 84) (Table 1).
b Diameter of the third ventricle.
  The mean diameter of the third ventricle in HD patients was 9.4 mm (range: 4–14.7 mm). A negative correlation was found between the diameter of the third ventricle and MMSE scores (Spearman rho − 0.37, 95% CI − 051 to − 0.2, *p* < 0.0001, Fig. 2Ba). A significant but weaker correlation was also present between the diameter of the third ventricle and TFC (Spearman rho − 0.26, 95% CI − 0.41 to − 0.08, *p* = 0.037, Fig. 2Bb). The diameter of the third ventricle was significantly smaller (5.6 mm, range: 2.5–8.9 mm) in the control group compared to HD patients (*t* test, *p* < 0.001) (Table 1).
c Echogenicity of the substantia nigra.
  The substantia nigra was bilaterally visualized in 119 HD patients. Altered echogenicity of the substantia nigra was found in 66.4% of patients (i.e., 79 patients) (Table 1). Among these patients, bilateral, marked hyperechogenicity was revealed in 21 patients (17.6%), unilateral marked hyperechogenicity in 37 patients (31.1%), bilateral medium hyperechogenicity in 40 patients (33.6%), and unilateral medium hyperechogenicity in 13 patients (10.9%) (Fig. 2Ca). There was a correlation between altered echogenicity UHDRS TMS values (Spearman rho 0.38, *p* < 0.001) (Fig. 2Cb). Moreover, there was a marked significant difference between the mean values of UHDRS TMS among patients in each groups, including bilateral marked echogenicity (50.4 ± 3.17, range 26–74, median 49, *n* = 21), unilateral marked echogenicity (40.7 ± 2.4, range 1–73, median 41, *n* = 37), bilateral medium hyperechogenicity (37.9 ± 8.2, range 9–83, median 35, *n* = 8), and unilateral medium hyperechogenicity (36.4 ± 3.7, range 1–50, median 42, *n* = 13). In the group of patients with bilateral normoechogenic substantia nigra, the lowest TMS value (28.9 ± 3.4, range 1–69, median 30, *n* = 40) was found (Kruskal–Wallis ANOVA *p* = 0.0057, KW = 14,56, Fig. 1b). In the control group, unilateral medium hyperechogenic substantia nigra was found in 9 out of 84 participants (i.e., 10.7%) (Table 1).
d Interrater agreement.
  The reliability of the raters’ assignment of patients to the aforementioned groups was assessed using the Kappa statistical test and showed a good agreement (i.e., kappa value: 0.712, SE: 0.083, 95% CI 0.550–0.874).

a Nucleus raphe echogenicity in HD patients with and without signs of depression.
  Altered echogenicity of the nucleus raphe (i.e., score 0 or 1) was found in 78% (i.e., 48 of 61) of patients with current signs of depression. In the group of patients with a previous history of depression, the proportion was lower—57% (i.e., 12 of 21 patients). A similarly lower rate was observed in the group of patients without depression (i.e., 56.8%, or 25 of 44 patients) (Fig. 2Aa) (For a summary see Table 1).

## Discussion

Our research highlights abnormalities in echogenicity of various deep brain structures in patients with HD.

a Nucleus raphe echogenicity.
  Decreased echogenicity of the serotoninergic nucleus raphe was found more frequently in HD patients than reported rates among healthy individuals. Indeed, in various reports, the percentage of hypoechogenic raphe in healthy, not depressed subjects varies from 8–9% (Walter et al 2007; Kostić et al. 2017) to 15% (Krogias et al 2011). In our study, abnormalities were present in 67% of HD patients; further, among HD patients, abnormalities were most frequently observed in patients with a current diagnosis of depression (i.e., 78%). Higher BDI depression scores were also positively correlated with the presence of raphe hypoechogenicity. This result is similar to a previous study by Krogias et al (2011), wherein the percentage of currently depressed patients, showing disturbed raphe echogenicity was 71.4%. Moreover, in Krogias et al. BDI and Hamilton Rating Scale for Depression (HAM-D) scores were significantly higher in HD subjects with disturbed raphe echogenicity. Interestingly, in contrast to findings of Krogias et al. in a relatively large group of patients without depression (including both patients successfully treated in the past and those without depressive episodes in their clinical history), the percentage of ultrasonographic abnormalities found in the raphe was high—around 57%. The pathophysiological basis of changes in midbrain raphe echogenicity in patients with depression still remains unclear. It is possible, thus, that lowered echogenicity reflects a decrease in both the cellular and fiber tracts density of this nucleus. On the other side, it is well known that dysfunction of the serotoninergic system is a feature not only of major depression but also depressive states in neurodegenerative disorders (Walter et al. 2007; Richter et al. 2018; Becker et al. 1997). In HD, dysregulation of the serotoninergic system has been well documented (Du et al. 2013; Paulsen et al. 2010) and results from basic cellular pathology. However, serotoninergic dysregulation is only one component that may contribute to the depressive state together with, for example, brain-derived neurotrophic factor (BDNF) depletion, hypothalamus–pituitary–adrenal (HPA) axis abnormalities, and significant environmental factors. This complexity may help to explain why abnormalities in the raphe nucleus were not found in all patients with depression in our study. On the other hand, the presence of raphe hypointensities in a significant percentage of patients without depression may reflect a susceptibility to the development of depression in the future. Interestingly, several other studies report that the lifetime rate of depression among HD patients can range from 30 to as high as 70% (Duff et al. 2007; Van Duijn et al. 2008). The latter percentage (i.e., 70%) is similar to the percent of HD patients in our study who showed decreased raphe echogenicity (i.e., 67%). Moreover, Krogias and Walter in a detailed review (2016) proved that the presence of hypoechogenic raphe is not depressed subjects both with and without a history of neurodegenerative disease is associated with a significantly higher risk of a future diagnosis of depression. From the clinical point of view, we could speculate, that revealing the raphe hypoechogenicity in not depressed HD subject may lead to a closer monitoring for depressive symptoms during future management, also when planning pharmacotherapy with agents that potentially may induce depressive states (like tetrabenazine).
b Diameter of the third ventricle.
  The assessment of the third ventricle is a simple and reliable measurement that correlates with brain volume loss in multiple sclerosis (Schminke et al. 2010), primary dementia (Wollenweber et al. 2011), PD, and atypical parkinsonian syndromes (Behnke et al. 2005). Given that atrophy of the brain parenchyma is a feature of HD, there should be a strong correlation between the diameter of the third ventricle and cognitive and functional decline, as measured by MMSE and TFC values. In our study, a correlation was present but not as strong as was expected based on measurements performed on a relatively large population. There are at least two possible explanations of this finding. First, studies performed during the Track-HD project (Tabrizi et al. 2013) revealed that the correlation between brain atrophy and cognitive and functional decline may not be linear. Indeed, HD pathology leads to an early progression of brain atrophy which can also induce compensatory mechanisms to delay the clinical presentation of cognitive decline. Second, another feature of HD is significant clinical variability, which leads to different disease presentations. This variability may leave cognitive functions relatively intact in some patients for a longer time than in others.
c Substantia nigra hyperechogenicity.
  Usefulness of substantia nigra TCS assessment in the diagnosis of PD has been proven in a number of studies (Walter 2009). However, some reports also describe disturbances in TCS presentation of the SN in other diseases, including multiple system atrophy, progressive supranuclear palsy, and corticobasal degeneration. For the latter disease, TCS disturbances are observed almost as frequently as in PD patients (Walter 2009). In an early study of basal ganglia echogenicity (Postert 1999), a hyperechogenic SN was found in 26% of HD patients; however, bilateral hyperechogenicity was detected only in 4.4%. The authors of that study found a correlation between the number of CAG repeats and the severity of clinical status. In a study by Krogias et al (2011), the percentage of patients with hyperechogenicity was significantly higher, and reached 41%, with about 10% HD patients with bilateral hyperechogenicity. However, a correlation between the presence of SN abnormalities and disease status was not found. Saft et al. (2015) found a higher frequency of SN hyperechogenicity among patients with juvenile HD (i.e., 100%) as compared to adult type (i.e., 29.3%). Importantly, in that study (Saft et al 2015), the presence of SN hyperechogenicity correlated with the bradykinesia subscore in the UHDRS, suggesting that these abnormalities may be a marker of the bradykinetic and rigid motor phenotype of HD. In our study, the percentage and degree of altered SN echogenicity were not only higher but was also correlated with the general motor status measured by the UHDRS TMS. Moreover, we found higher values of TMS in patients with bilateral marked hyperintensity of SN.

The pathogenic basis of altered SN echogenicity is still speculative. However, one of the most likely hypotheses assumes that SN echogenicity is caused by abnormal accumulation of metals in the basal ganglia. This mechanism is described in PD (Berg et al 2002), but iron accumulation in the basal ganglia, in particular, has also been reported in HD [for a review, see Muller and Leavitt (2014)] and correlates with HD patient’s motor status (Jurgens et al. 2010). Other theories connect SN changes with a decrease in cellular density and gliosis. All of these mechanisms are present in HD and undergo progression with the course of the disease, which may be a possible explanation for the observed correlation between SN ultrasonographic presentation and severity of motor symptoms.

## Conclusions

In the present study, a TCS procedure was added to routine follow-up visits for patients in our HD center.

Our results reveal a correlation between some TCS findings and clinical status among HD patients, including motor signs and psychiatric symptoms. The simplicity and availability of TCS examinations makes it an attractive additional tool for tracking disease progression.

## Data Availability

Blinded data in.xls formats as well as blinded and encoded DICOM images are available upon requests.

## Abbreviations

HD: Huntington’s disease
BDI: Beck’s Depression Inventory
MMSE: Mini-Mental State Examination
TFC: Total functional capacity
UHDRS: Unified Huntington’s Disease Rating Scale
TMS: Total motor score
SN: Substantia nigra
TCS: Transcranial sonography
